# International experiences during United States ophthalmology residency training: Current structure of international experiences and perspectives of faculty mentors at United States training institutions

**DOI:** 10.1371/journal.pone.0225627

**Published:** 2019-11-26

**Authors:** Mona L. Camacci, Tara E. Cayton, Michael C. Chen

**Affiliations:** Department of Ophthalmology, Penn State College of Medicine, Hershey, Pennsylvania, United States of America; Centers for Disease Control and Prevention, UNITED STATES

## Abstract

**Background:**

There is a high level of interest in international experiences during United States (U.S.) ophthalmology residency training among both program directors and trainees.

**Methods:**

An electronic invitation to a 26-question survey was sent to all 114 U.S. ophthalmology residency program directors. The invitation requested that the survey be completed by the one faculty member who was most involved in overseeing the international experiences for the residents. The survey consisted of multiple choice and Likert-type scale questions. The Mann-Whitney U test was used for analysis of demographic data and Friedman’s test and Wilcoxon-Signed Rank test were used to analyze ranked responses.

**Results:**

Responses were obtained from 70 faculty mentors representing unique programs, yielding a response rate of 61.4%. The majority of programs that responded (88.6%, n = 62) either offered international ophthalmology experiences for residents or supported residents finding their own experiences to go abroad. International experience participation rate among residents correlated with the number of years the experiences had been offered by the programs (p = 0.001). More than half of the respondents (55.0%, n = 33) felt that the residents benefited more than the hosts during these international experiences. Approximately half of the respondents (51.6%, n = 32) believed that additional training beyond what is covered in the standard curriculum to practice ophthalmology in the U.S. is necessary for practicing ophthalmology in an international setting.

**Conclusions:**

There is high interest and participation in international experiences within U.S. ophthalmology residency programs. This high participation warrants further investigation into the long-term impact of these international experiences and how U.S. residency programs can structure these experiences to maximize the benefits to both the residents and the international host communities.

## Introduction

International health electives offered by United States (U.S.) residency programs are becoming increasingly popular among trainees [1–5]. International health electives raise awareness regarding the cultural and socioeconomic factors that affect health [6–8]. In addition, these electives also cultivate the core competencies determined by the Accreditation Council for Graduate Medical Education (ACGME), including patient care, medical knowledge, practice-based learning and improvement, systems-based practice, professionalism, and interpersonal skills and communication [9–13]. Concurrently, there is a growing need for international surgery training as studies have shown that morbidity and mortality rates have increased in low income countries due to limited access to basic surgical care [14–16].

Given the amount of preventable and treatable blindness worldwide, ophthalmology lends itself to global work [17–20]. Not surprisingly, there is high interest in the topic of international ophthalmology in U.S. residency programs. A study conducted by Coombs et al. found that 83% of programs represented in their survey participated in global health, with 54% of residency programs developing international electives [5]. Similarly, a recent survey of applicants to U.S. ophthalmology residency programs showed that nearly all respondents (95.4%) reported an interest in participating in an international experience during residency [21].

Given the significant interest and participation in international ophthalmology among U.S. ophthalmology residency programs, it is important to explore the structure of these experiences to assess how to maximize the benefits to the residents and the international communities that host the residents, to better inform not only residency training in ophthalmology but also in other medical specialties. This study aims to analyze the current structure of international experiences in U.S. ophthalmology residency programs and to explore the perspectives of international experiences from the standpoint of the faculty mentors who oversee these experiences.

## Methods

An anonymous, cross-sectional survey of all 114 U.S. ophthalmology residency programs participating in the 2018–2019 San Francisco (SF) Match was conducted from June to July 2018. REDCap electronic data capture tool hosted at Penn State Hershey Medical Center was used to collect and manage the study data [22]. Survey questions were developed based on questions from previously published international ophthalmology surveys [5,21] as well as new questions designed with the input of faculty, residents, and medical students at the Penn State College of Medicine involved in international health. The survey was pilot tested on nine faculty members, representing six academic departments within the Penn State College of Medicine, who had personal experience in international health and/or experience supervising medical trainees in international health experiences. The survey questions were finalized after incorporating the feedback from the pilot testing. An electronic invitation was emailed to the residency program director at each U.S. program, as listed on the SF Match or Fellowship and Residency Electronic Interactive Database (FREIDA). The email requested that the survey be completed by the one faculty member, as identified by the program director, who was most involved in overseeing the international experiences for the residents. While in many cases this individual was likely to be the program director, in the case where this was not, the program director was asked to forward the survey invitation to the designated faculty member. Respondents anonymously completed a 26-item questionnaire which included multiple choice and Likert-type scale questions (S1 Appendix). One reminder email was sent two weeks after the initial email. A paper version of the survey was additionally sent to the programs that had not responded to the electronic survey invitation.

Statistical analysis was performed using SPSS (Version 25.0 IBM Corp. Armonk, NY). For the purpose of statistical comparison, ophthalmology programs were divided into two groups based on the number of years international experiences had been offered for the residents. Programs that had offered international ophthalmology experiences for six or more years were designated as “established programs” and programs that had offered international ophthalmology experiences for five or fewer years were designated as “newer programs.” The Mann-Whitney U test was used for analysis of demographic data and to assess the relationship of the ranked responses between independent groups. Friedman’s test and Wilcoxon-Signed Rank test were used to analyze ranked responses. Missing information was handled by a pairwise deletion for each analysis completed. P-values < 0.05 were considered statistically significant for all analyses, except when the Bonferroni correction was applied to multiple simultaneous comparisons. This study was deemed exempt by the Penn State Hershey Institutional Review Board.

## Results

**Fig 1:**
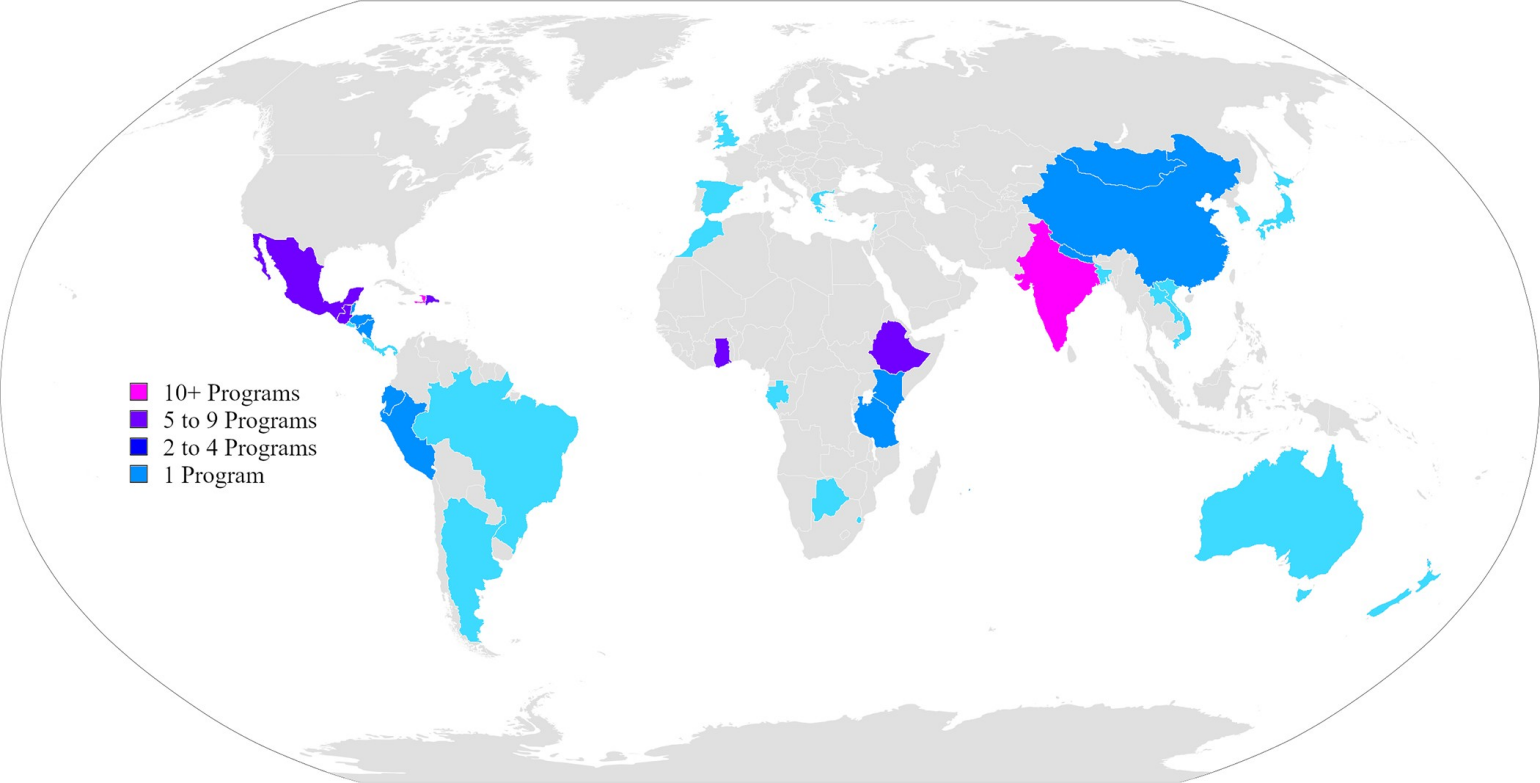
International ophthalmology host countries. A world map showing the countries to which U.S. ophthalmology residents have traveled over the last five years. Each of the identified countries is color-coded according to the number of times it was reported by the respondents. For list of countries, please reference supplemental materials (S2 Appendix). (Image adapted from Canuckguy. *BlankMap-World6*. Public Domain. 2006. https://commons.wikimedia.org/wiki/File:BlankMap-World.svg. Accessed August 21, 2018).

Responses were received from 70 faculty mentors representing unique residency programs, yielding a response rate of 61.4% (S3). Seven surveys contained missing responses to some of the questions. Of those who answered the question, 89.1% (n = 49) were program directors. Of the residency programs represented, 88.6% (n = 62) either offered international ophthalmology experiences for residents or supported residents finding their own experiences to go abroad. The vast majority of programs that supported international ophthalmology experiences (87.1%, n = 54) reported resident participation in international experiences within the past three years. Respondents reported a total of 41 different host countries to which residents have traveled to internationally over the last five years, with India (n = 18) and Haiti (n = 11) being the most commonly reported countries (Fig 1).

### Comparison of established programs and newer programs

The demographics of the residency programs supporting international experiences are summarized in (Table 1). Compared to newer programs, established programs reported higher rates of resident travel within the last three years, faculty travel to the international site(s), sending residents to specific sites at least annually, offering a formal elective, and hosting international trainees or faculty. Compared with newer programs, a higher percentage of established programs offered department funding and did not require the use of resident vacation time for the international experience. The most common international experience duration for both established and newer programs was one week or less.

**Table 1:** Demographics of newer programs^a^ and established programs^b^.

	Total Programs (n = 62)	Newer Programs (n = 32)	Established Programs (n = 30)	
	%	%	%	P-value
**Resident Participation Within Last 3 Years**				
Mean	32.6 ± 32.5	21.5 ± 26.4	44.4 ± 34.6	0.001*
Median	20.0	10.0	31.5	
Range	0–100	0–90	0–100	
**Faculty Travel to Sites**				
Yes	35.5	18.8	53.3	0.005*
No	64.5	81.3	46.7	
**Residents Sent to Sites at Least Annually**				
Yes	40.3	28.1	53.3	0.045
No	59.7	71.9	46.7	
**Formal International Ophthalmology Elective**				
Yes	33.9	25.0	43.3	0.131
No	66.1	75.0	56.7	
**Length of International Experience**				
≤ 1 week	55.7	68.8	41.4	0.063
2 weeks	26.2	15.6	37.9	
3 weeks	6.6	6.3	6.9	
4 weeks	11.5	9.4	13.8	
**Vacation Time Use for International Experience**
Required	32.3	43.8	20.0	0.012
Partially required	21.0	25.0	16.7	
Not required	46.8	31.3	63.3	
**Funding for International Experience**				
Department funding available	59.7	46.9	73.3	0.035
Department funding not available	40.3	53.1	26.7	
**Hosting of International Trainees/Faculty**				
Yes	60.0	43.8	78.6	0.006*
No	40.0	56.3	21.4	

* Statistically significant p-values, obtained using Mann-Whitney U test, are shown with an asterisk. Statistical significance was defined as p<0.0063, based on the Bonferroni correction for multiple comparisons.

^a^offered international experiences five years or fewer

^b^offered international experiences six years or more

### Resident responsibilities

When asked about resident responsibilities to the home institution before and after participating in an international experience, 40.3% (n = 25) of respondents reported having no mandatory responsibilities before participating in an international experience and 25.8% (n = 16) of respondents reported no mandatory responsibilities after returning. Of the programs that did have requirements, the most commonly reported requirements before participating in an international experience included a formal meeting with a faculty mentor (81.1%, n = 30), creation of personalized learning objectives (37.8%, n = 14), and surgical wet lab (37.8%, n = 14). Requirements after returning from an international experience included formal presentation (78.3%, n = 36), debriefing with faculty mentor (41.3%, n = 19), and written reflection on experience (23.9%, n = 11).

Respondents who reported specific sites where they send residents at least annually (n = 25) were asked who their residents work with most during an international experience. The most common response was local ophthalmologists from the host site (48.0%, n = 12), followed by ophthalmologists from the U.S. (40.0%, n = 10).

### The need for additional training

**Fig 2:**
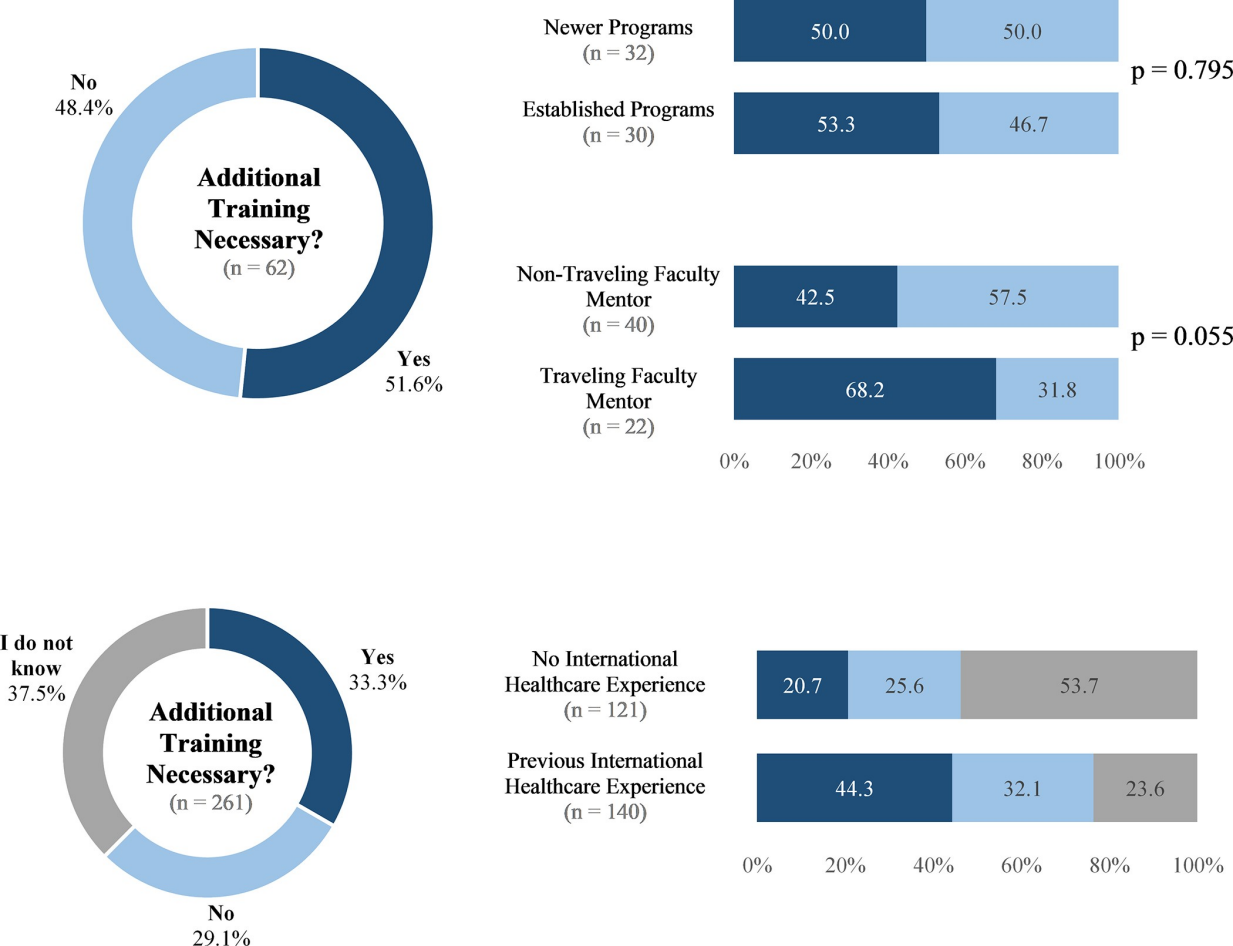
Views on additional training. Responses to the survey question, “Do you think additional training (beyond what is covered in the standard curriculum to practice ophthalmology in the United States) is necessary to practice ophthalmology in an international setting?” Figure on the left depicts responses of both subgroups combined, and figures on the right depict responses of each subgroup. P-values were generated using Mann-Whitney U test analysis. Statistical significance was defined as p<0.025, based on the Bonferroni correction for multiple comparisons.

**Fig 3:**
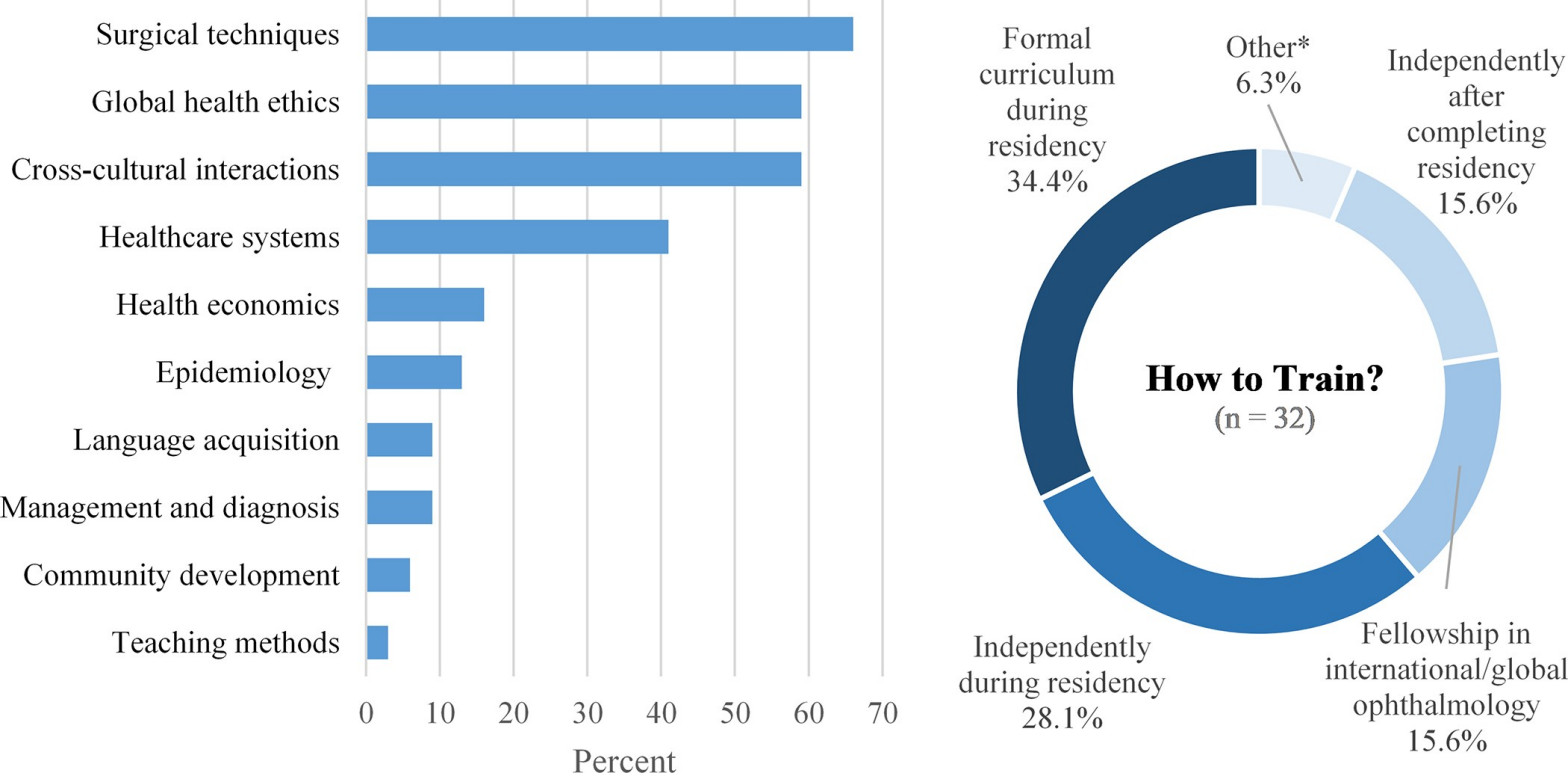
International ophthalmology curriculum. Figure on the left depicts answers from respondents who answered that additional training is necessary to the survey question, “What type of additional training is most needed? Please select your top three choices.” (n = 32). Figure on the right depicts answers from respondents who answered that additional training was needed to the survey question, “How should this additional training be obtained?” (n = 32). *Other write-in responses: “Surgical electives and the integration of material into formal residency didactics” and “Basic curriculum to all residents during residency. Additional training for those who are interested in going abroad independently”.

Roughly half of the respondents (51.6%, n = 32) believed that additional training beyond what is covered in the standard curriculum to practice ophthalmology in the U.S. is necessary to practice ophthalmology in an international setting (Fig 2). The proportion of respondents with this view was similar between both the newer and established programs. However, though not statistically significant, faculty from both newer and established programs who had traveled to any of the same sites as their residents were more likely to respond that additional training is necessary (68.2%, n = 15 versus 42.5%, n = 17, p = 0.055). Of the respondents who believed additional training is necessary, the topics identified by more than half of respondents included surgical techniques (65.6%, n = 21), cross cultural interactions (59.4%, n = 19), and global health ethics (59.4%, n = 19, Fig 3). When asked how this additional training should be obtained, the most common response was through a formal curriculum during residency.

### Benefits of international experience

**Fig 4:**
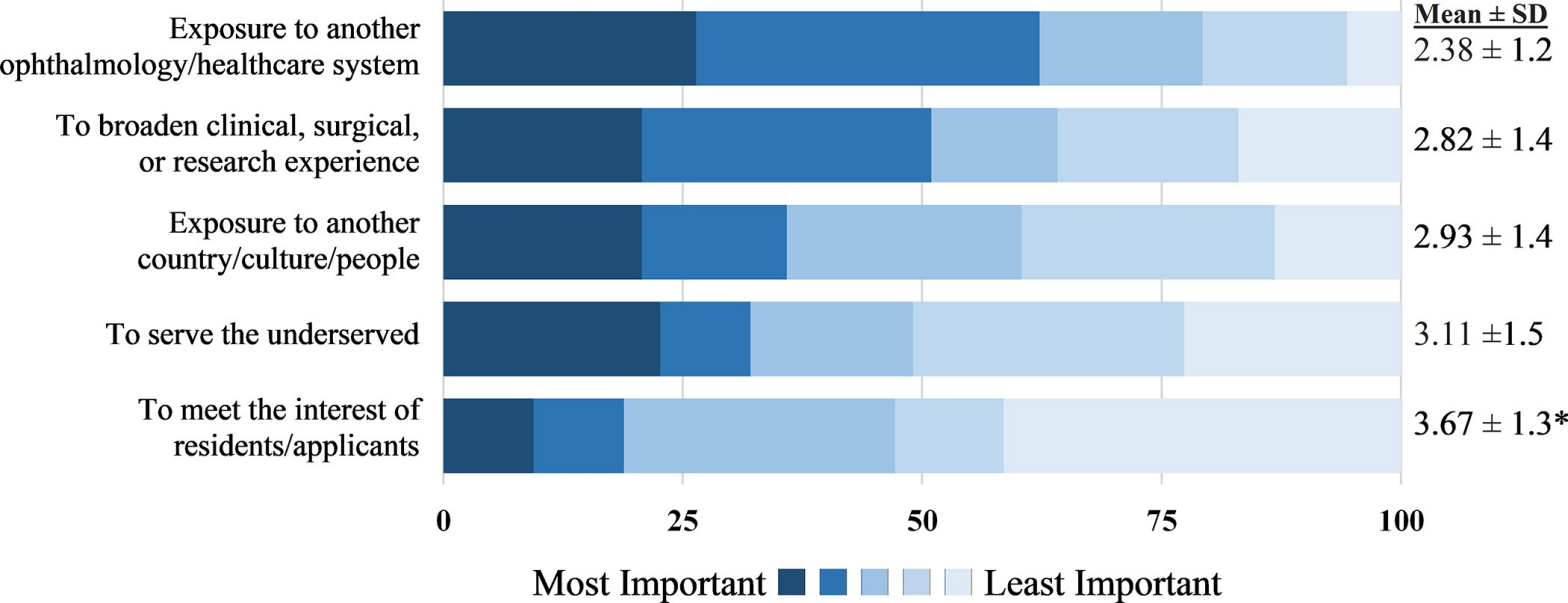
Goals of international ophthalmology experience. Responses to the survey question, “Why do you think it is important that international experiences be made available to your residents? Please rank the following choices from 1 (most important) to 5 (least important).” Percentage of respondents, mean, and standard deviation shown (n = 54). Statistically significant p-values, obtained using Mann-Whitney U test, are shown with an asterisk, comparing responses from “newer” and “established” programs. Statistical significance was defined as p<0.01, based on the Bonferroni correction for multiple comparisons. *To meet interest of residents, “newer versus “established” programs (mean rank 3.07 versus 4.43 respectively; p = 0.0003).

When asked why it was important that international experiences be made available to residents, “Exposure to another ophthalmology/healthcare system,” “To broaden clinical, surgical, or research experience,” and “Exposure to another country/culture/people” outranked “To serve the underserved” (Fig 4).

**Fig 5:**
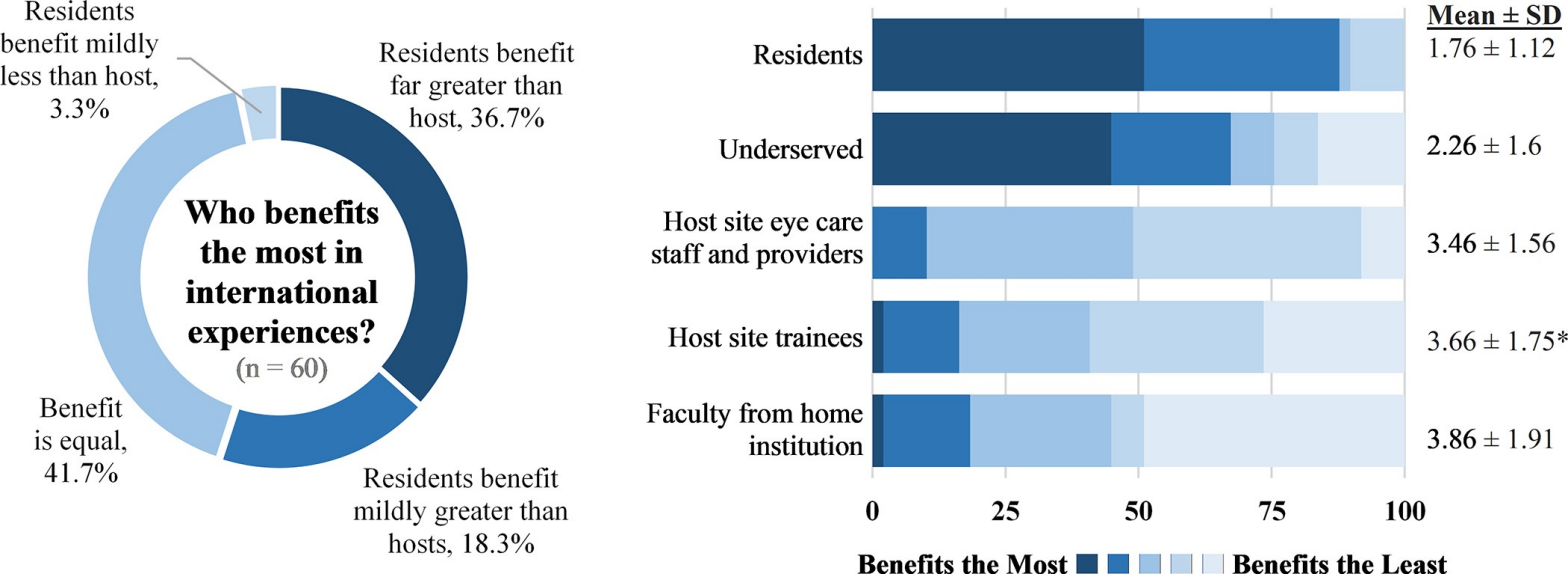
Beneficiaries of international ophthalmology experience. Figure on the left depicts responses to the survey question, “Regarding international experiences and the benefits to your residents and international hosts, with which of these statements do you most agree?” (n = 60). Figure on the right depicts responses to the survey question, “Different parties may benefit from the global health endeavors of your institution. From your perspective, which parties benefit the most, and which parties benefit the least? Please rank the parties from 1 (benefits the most) to 5 (benefits the least) and leave the ranking blank if these parties do not exist in your arrangement.” Percentage of respondents, mean, and standard deviation shown for those who ranked all categories (n = 50). Statistically significant p-values, obtained using Mann-Whitney U test, are shown with an asterisk, comparing respondents from “newer” and “established” programs. Statistical significance was defined as p<0.01, based on the Bonferroni correction for multiple comparisons. *Host site trainees, “newer” versus “established” programs, (mean rank 3.26 versus 4.13 respectively; p = 0.005).

More than half of respondents (55.0%, n = 33) felt that the benefit to the residents was greater than the benefit to the international host, and 41.7% (n = 25) felt the benefit was equal (Fig 5). Only 3.3% (n = 2) felt that the benefit to the residents was less than the benefit to the international host. These views were similar between respondents from established programs and from newer programs (p = 0.573). Benefits to the residents outranked benefits to the underserved patient populations, host site eye-care staff and providers, and host site trainees (Fig 5).

### Hosting of international faculty and trainees

Of the respondents who either offered international ophthalmology experiences for residents or supported residents finding their own international experiences, 60.0% (n = 36) hosted international trainees or faculty at their home institutions (Table 1). Established programs were more likely than newer programs to host international visitors (78.6%, n = 22 versus 43.8%, n = 14, p = 0.006). Those hosted included physicians who have completed training (80.6%, n = 29), residents and fellows (63.9%, n = 23), and medical students (27.8%, n = 10). Of those hosted, 19.4% (n = 7) were from the same international sites attended by the U.S. residents.

## Discussion

**Fig 6:**
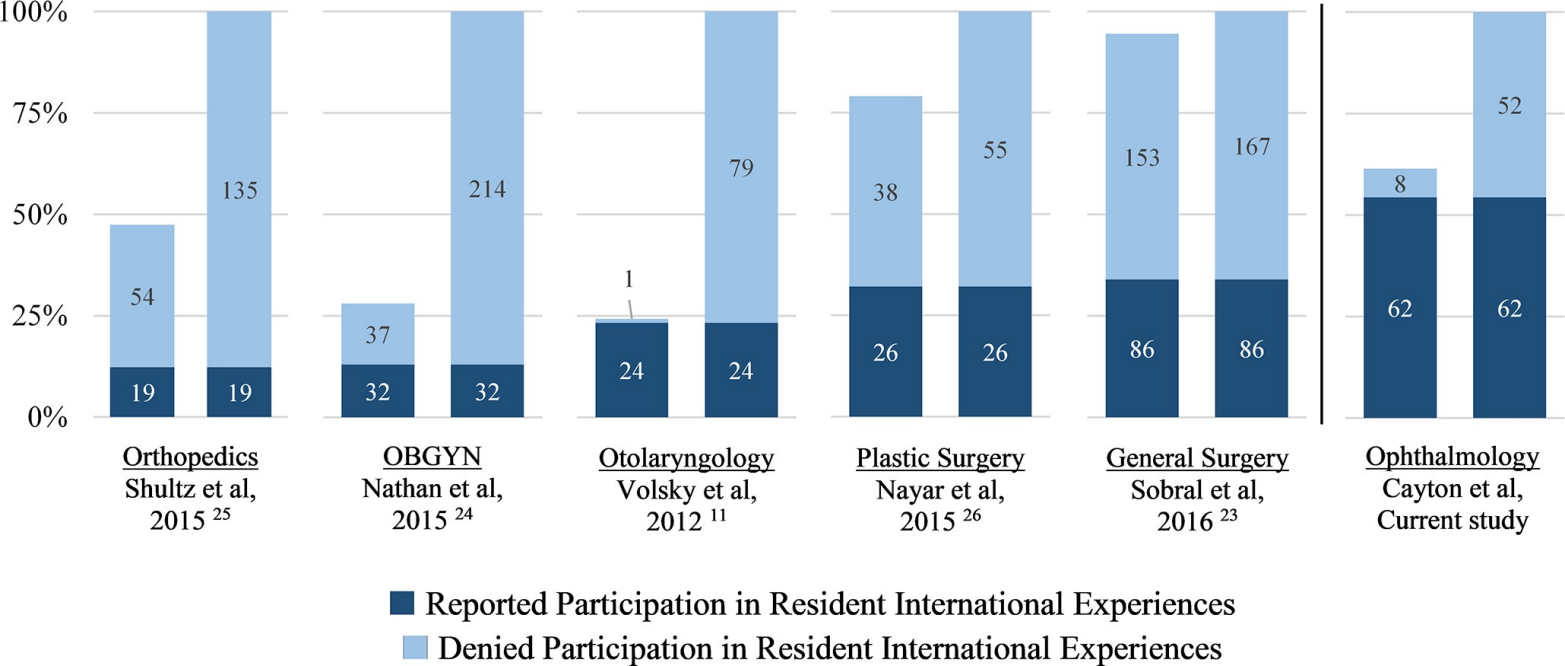
Comparison of survey responses for participation in resident international experiences. For each surgical specialty, the left bar represents the responses reporting resident participation in international experience during training. The overall response rate for each survey is represented by the vertical axis. For the purpose of comparison across studies, the bar on the right for each specialty represents what participation rates would theoretically look like under the extreme hypothetical scenario if all survey non-responders denied participation in international experiences for residents.

Previous studies have suggested that interest in international ophthalmology is high among both U.S. residency program directors and applicants [5,21]. This study further confirms this sentiment by showing that not only is interest high, but participation in international experiences is also high, as 88.6% of ophthalmology residency programs represented in this study offer or support international experiences. Recent surveys of U.S. residency programs have shown that many surgical specialties offer international experiences during residency training: orthopedic surgery (26.0%), general surgery (36.0%), plastic surgery (40.6%), OBGYN (46.4%), and otolaryngology (96.0%) (Fig 6) [11,23–26]. As the response rate of these studies vary considerably between 24.3% and 94.5%, it is difficult to compare the program participation rate in this study with the participation rates of residencies of other specialties. Taking the extreme hypothetical scenario that the ophthalmology residency programs not captured in this survey do not offer or support international experiences, more than half of U.S. ophthalmology residency programs (54.4%) still support these experiences. Applying this same extreme scenario to the studies from other surgical specialties, the participation rate within U.S. ophthalmology residency programs in this study appears considerably higher than that in U.S. residency programs of other surgical specialties (Fig 6). This high participation may be related to the fact that the majority of causes of visual impairment worldwide are either preventable or treatable in a cost-effective manner [27]. For example, cataract surgery, one of the most commonly performed surgeries in the world, has characteristics of an ideal surgical procedure to be performed in a low-resource setting [28].

### Beneficiaries of international experiences

The majority of respondents felt that residents benefited from the international experience more than the international hosts. Faculty mentors ranked resident exposure and education categories significantly higher than the opportunity to serve the underserved. Within a residency training paradigm where education is a primary goal, it may be expected that the residents would be the primary beneficiaries of an international experience rather than the other parties involved, including the hosts. Interestingly, this majority perspective among faculty mentors differs from the majority perspective of ophthalmology residency applicants in a previous study, where 59.0% of respondents ranked “offer service to the underserved” as the most important goal of an international experience, over learning goals such as “learn from national eye care provider(s) in the country visited” and “learn about another eye care system” [21]. This suggests that there exists a disconnect between the goals and objectives that faculty mentors intend for residents and the trainee expectations of what an international experience will provide. Addressing the goals and expectations for an international experience may help trainees develop a better understanding of their work in an international setting.

Although there currently is no literature on the perspectives of international hosts that work with U.S. ophthalmology trainees, the notion that the international hosts may benefit less than the residents should be evaluated in light of recent literature from other specialties that highlight the perspectives of international hosts in other medical fields [29–32]. Imbalanced partnerships have the potential to result in issues of power inequality, unethical approaches to clinical resource limitations, unsustainable projects, and exploitation of host faculty and resources [29,31]. In particular, international hosts have cited cultural differences, differing expectations, and miscommunication as issues that lead to inappropriate care of patients [30,32]. These studies have provided key suggestions to improve and foster reciprocity surrounding international experiences, including creating mutually agreed upon goals, having trainees actively learn about the cultural and environmental context before visiting, and working towards formal, long-term, sustainable partnerships [29–32]. As more of the residency programs in this study categorized as “established,” compared with the residency programs that were categorized as “newer,” reported having a formal elective, hosting international trainees or faculty, faculty travel to the international site(s), and sending residents to specific sites at least annually, this may suggest that as residency programs become more experienced with sending trainees internationally that they may be intentionally developing their programs to be more sustainable and of mutual benefit both to the residents and international hosts.

### Opportunities to shape international residency education

Almost half of the faculty mentors surveyed do not believe any additional training is necessary to practice ophthalmology internationally beyond what is covered in the current residency curriculum. A similar number of the programs (40%) require no mandatory education responsibilities before residents travel internationally. Although not statistically significant, faculty mentors who had traveled to the international host sites were more likely to believe that additional training is necessary as compared to those who had not. These findings are similar to the findings of a previous study in which ophthalmology applicants who had previously participated in a healthcare-related experience in an international setting were more likely to believe that additional training was necessary [21]. The healthcare system at an international site may differ in aspects such as resource availability, healthcare coverage, socioeconomics, cultural factors, clinical pathologies, and surgical techniques. It is possible that faculty mentors and applicants who have traveled internationally have more awareness of complexities that may necessitate a different approach to what is taught in the standard U.S. residency curriculum.

Although ophthalmology residents can receive credit for time spent internationally,[33] as it stands, there is no required curriculum for residents taking part in international experiences. The academic ophthalmology community has a great opportunity to create a structured curriculum that encourages residents to continue the practice of thoughtful international health collaboration throughout their entire career. This can further be extrapolated to all medical specialties as they continue to explore global health education and find ways to maximize benefit both for the trainees and the host partners.

### Limitations

There were several limitations in this study, one being the partial response rate. This being said, the response rate of 61.4% is comparable to the response rates of previous studies that surveyed residency program directors of ophthalmology and other surgical specialties [5,11,23–26,34,35]. Inherent to surveys with partial response rates, there may be a response bias, with faculty mentors at institutions that offer international experiences more likely to respond than those at institutions that do not. The authors recognize that the survey mechanism in this study, consisting of multiple choice and Likert-scale type questions, was not able to capture every possible scenario involving international experiences in residency programs. Among the feedback received from the respondents, one respondent commented that as their program sent residents to different sites with different structural arrangements, the answers to the questions depended on which site was in mind when answering the survey. Another respondent commented that despite the fact that their residency program had offered international experiences to their residents for more than twenty years, they no longer support residents going abroad due to policies from their graduate medical education department. The findings of this study, including the feedback received, suggest that further investigation using other information gathering methods may be beneficial.

## Conclusion

In conclusion, there is high interest and participation in international experiences in U.S. ophthalmology residency programs. Although this study was performed on ophthalmology residency programs, there are aspects of global health education that are not specialty specific, and some of the findings and implications of this study may be applicable to residency programs in other medical and surgical specialties. There is great opportunity for U.S. residency programs to work with international hosts in determining how to structure these international experiences and to shape pre- and post-experience education, not only to maximize the benefits to the residents, but also to the host communities.

## Supporting information

S1 Appendix Anonymous questionnaire. A 26-item questionnaire including multiple choice and Likert-type scale questions.
(DOCX)

S2 Appendix List of international ophthalmology host countries. List displaying all countries respondents listed as countries their residents have visited in the last five years.
(DOCX)

S3 Appendix Collected data. Complete collection of data from anonymous questionnaire of 70 faculty mentors.
(XLSX)
