# Supplementary material for: International experiences during United States ophthalmology residency training: Current structure of international experiences and perspectives of faculty mentors at United States training institutions
Source: PLoS One. 2019 Nov 26;14(11):e0225627. doi: 10.1371/journal.pone.0225627 (PMC6879160; doi:10.1371/journal.pone.0225627)
Supplement: S1 Appendix — A 26-item questionnaire including multiple choice and Likert-type scale questions. (DOCX) [file pone.0225627.s001.docx]

**Ophthalmology International Experiences Survey**

**Supplemental 1 (S1)**

**1. How many years has your residency program offered international ophthalmology experiences for your residents?**

*Select one.*

◯ 1 year or less

◯ 2-5 years

◯ 6-10 years

◯ 10+ years

◯ We do not offer international experiences through our program, but are supportive of residents finding their own experiences to go abroad

◯ We do not support residents going abroad during their residency training

**1a. Does your program have a formal international ophthalmology elective?**

◯ Yes

◯ No

**2. How is the international experience funded for residents?**

*Select all that apply.*

◯ Department/Institutional funding

◯ Residents must find funding on their own

◯ Other

**2a. Is the department/institutional funding designated specifically for international ophthalmology experiences?**

◯ Yes

◯ No

**3. Are residents required to use vacation time for the international experience?**

*Select one.*

◯ Yes, residents must use vacation time for the entirety of the international experience

◯ Yes, residents must use vacation time for a portion of the international experience

◯ No, residents are not required to use vacation time

**4. What percentage of your residents have participated in an international experience within the last 3 years?**

**5. Have you traveled to any of the sites that your residents have visited?**

◯ Yes

◯ No

**5a. Have you traveled to any of the sites that your residents have visited within the past 3 years?**

◯ Yes

◯ No

**6. Are there specific sites that you send your residents to at least annually?**

◯ Yes

◯ No

**6a. Which countries do your residents go to at least annually?**

*Please list all countries.*

**6b. Which countries have your residents gone to in the last 5 years?**

*Please list all countries.*

**6c. Select who your residents work with MOST during an international experience: *Select one.***

◯Local ophthalmologists from the host site

◯ Local eye care providers (non-ophthalmologists) from the host site

◯ Non-eye care medical providers from the host site

◯ Ophthalmologists from the United States

◯ Non-eye care medical providers from the United States

◯ Other

**7. How long are the majority of your international experiences?**

*Select one*

◯ 1 week

◯ 2 weeks

◯ 3 weeks

◯ 4 weeks

◯ Other

**8. Do you think additional training (beyond what is covered in the standard curriculum to practice ophthalmology in the United States) is necessary to practice ophthalmology in an international setting?**

◯ Yes

◯ No

**8a. What type of additional training is most needed?**

*Please select your top three choices.*

◯ The diagnosis and management of clinical disease

◯ Surgical techniques

◯ Teaching methods

◯ Healthcare systems

◯ Cross -cultural interactions

◯ Language acquisition

◯ Global health ethics

◯ Community development

◯ Health economics

◯ Epidemiology

◯ Other

**8b. How should this additional training be obtained?**

*Select one.*

◯Formal curriculum during residency

◯ Independently during residency

◯ Fellowship in international/global ophthalmology

◯Independently after completing residency

◯ Other

**9. What responsibilities do your residents have to their home institution before participating in an international experience?**

*Select all that apply.*

◯ Lectures

◯ Online modules

◯ Surgical wet lab

◯ Meeting with faculty mentor

◯ Creation of personalized learning objectives

◯ No mandatory responsibilities

◯ Other

**9a. What responsibilities do your residents have to their home institution after returning from an international experience?**

*Select all that apply.*

◯ Formal presentation

◯ Debriefing with faculty mentor

◯ Clinical logs

◯ Written narrative/reflection on experience

◯ Clinical/surgical logs

◯ No mandatory responsibilities

◯ Other

**10. Regarding international experiences and the benefits to your residents and international hosts, with which of these statements do you MOST agree?**

*Select one.*

◯ The benefit to the residents is far greater than the international host's benefit

◯ The benefit to the residents is mildly greater than the international host's benefit

◯ The benefit to the residents is equal to the international host's benefit

◯ The benefit to the residents is mildly less than the international host's benefit

◯ The benefit to the residents is far less than the international host's benefit

**11. In the last five years, has your home institution hosted international trainees or faculty?**

◯ Yes

◯ No

**11a. Are these trainees/faculty from the same international sites that your residents go to?**

◯ Yes

◯ No

**11b. Which levels are the trainees/faculty that come to your home institution*?*** *Select all that apply.*

◯ Medical students

◯ Trainees including residents and fellows

◯ Physicians who have completed training

◯ Other

**12. Why do you think it is important that international experiences be made available to your residents?**

*Please rank the following choices from 1 (most important) to 5 (least important).*

| Rank # |  |
| --- | --- |
|  | To provide residents exposure to another country/culture/people |
|  | To meet the interest of residents/applicants |
|  | To provide residents exposure to another ophthalmology/healthcare system |
|  | To provide residents the opportunity to broaden clinical, surgical, or research experience |
|  | To provide residents the opportunity to serve the underserved |

**13. Different parties may benefit from the global health endeavors of your institution. From your perspective, which parties benefit the most, and which parties benefit the least?**

*Please rank the parties from 1 (benefits the most) to 5 (benefits the least) and leave the ranking blank if these parties do not exist in your arrangement.*

| Rank # |  |
| --- | --- |
|  | Underserved patient populations in the vicinity of the host site |
|  | Residents from home institution |
|  | Faculty from home institution |
|  | Host site eye-care staff and providers |
|  | Host site trainees |

**14. Are you the residency program director?**

◯ Yes

◯ No

**15. May we contact you if we have further questions?**

◯ Yes

◯ No
