# Supplementary material for: International experiences during United States ophthalmology residency training: Current structure of international experiences and perspectives of faculty mentors at United States training institutions
Source: PLoS One. 2019 Nov 26;14(11):e0225627. doi: 10.1371/journal.pone.0225627 (PMC6879160; doi:10.1371/journal.pone.0225627)
Supplement: S2 Appendix — List displaying all countries respondents listed as countries their residents have visited in the last five years. (DOCX) [file pone.0225627.s002.docx]

**Supporting Material 2: List of International Ophthalmology Host Countries**

| **Country** | **Number of US Residency Programs who sent residents to site in last 5 years** |
| --- | --- |
| American Samoa | 1 |
| Antigua and Barbuda | 1 |
| Argentina | 1 |
| Australia | 1 |
| Bangladesh | 1 |
| Belize | 3 |
| Botswana | 1 |
| Brazil | 1 |
| China | 2 |
| Costa Rica | 1 |
| Dominican Republic | 5 |
| Ecuador | 3 |
| El Salvador | 1 |
| Ethiopia | 5 |
| Gabon | 1 |
| Ghana | 5 |
| Greece | 1 |
| Guatemala | 7 |
| Haiti | 11 |
| Honduras | 3 |
| India | 18 |
| Japan | 1 |
| Kenya | 3 |
| Laos | 1 |
| Lebanon | 1 |
| Mauritius | 1 |
| Mexico | 7 |
| Mongolia | 2 |
| Morocco | 1 |
| Myanmar | 1 |
| Nepal | 2 |
| New Zealand | 1 |
| Nicaragua | 3 |
| Panama | 1 |
| Peru | 4 |
| South Korea | 1 |
| Spain | 1 |
| Swaziland | 1 |
| Tanzania | 2 |
| United Kingdom | 1 |
| Vietnam | 1 |
